# Higher Neuronal Facilitation and Potentiation with APOE4 Suppressed by Angiotensin II

**DOI:** 10.21203/rs.3.rs-2960437/v1

**Published:** 2023-05-26

**Authors:** Sarah B. Scheinman, Kuei Y. Tseng, Simon Alford, Leon M Tai

**Affiliations:** University of Illinois at Chicago College of Medicine; University of Illinois at Chicago College of Medicine; University of Illinois at Chicago College of Medicine; University of Illinois at Chicago College of Medicine

**Keywords:** APOE4, hippocampus, neuron activity, angiotensin II

## Abstract

Progressive hippocampal degeneration is a key component of Alzheimer’s disease (AD) progression. Therefore, identifying how hippocampal neuronal function is modulated early in AD is an important approach to eventually prevent degeneration. AD-risk factors and signaling molecules likely modulate neuronal function, including *APOE* genotype and angiotensin II. Compared to *APOE3*, *APOE4* increases AD risk up to 12-fold, and high levels of angiotensin II are hypothesized to disrupt neuronal function in AD. However, the extent that *APOE* and angiotensin II modulates the hippocampal neuronal phenotype in AD-relevant models is unknown. To address this issue, we used electrophysiological techniques to assess the impact of *APOE* genotype and angiotensin II on basal synaptic transmission, presynaptic and post-synaptic activity in mice that express human *APOE3* (E3FAD) or *APOE4* (E4FAD) and overproduce Aβ. We found that compared to E3FAD mice, E4FAD mice had lower basal synaptic activity, but higher levels of paired pulse facilitation (PPF) and Long-Term Potentiation (LTP) in the Schaffer Collateral Commissural Pathway (SCCP) of the hippocampus. We also found that exogenous angiotensin II has a profound inhibitory effect on hippocampal LTP in both E3FAD and E4FAD mice. Collectively, our data suggests that *APOE4* and Aβ are associated with a hippocampal phenotype comprised of lower basal activity and higher responses to high frequency stimulation, the latter of which is suppressed by angiotensin II. These novel data suggest a potential mechanistic link between hippocampal activity, *APOE4* genotype and angiotensin II in AD.

## Introduction

Alzheimer’s disease (AD) is a progressive neurodegenerative disorder characterized by progressive learning and memory impairment [1, 2]. The hippocampus is one of the brain regions most affected in AD patients with high levels of Aβ plaques and neurofibrillary tangles and extensive neuronal atrophy [3–5]. Hippocampal neuronal dysfunction in AD is likely progressive, starting with altered glutamatergic activity and connectivity, culminating in cell death. Increasingly recognized is the importance of identifying how hippocampal neuronal function is modulated early in AD, to eventually prevent degeneration. A key component is understanding the impact of known AD-risk factors and signaling molecules on the hippocampal glutamatergic activity phenotype, here we focus on *APOE* and angiotensin II.

*APOE* is the greatest genetic risk factor for sporadic AD, with *APOE4* increasing risk up to 12-fold compared to *APOE3* [6, 7]. In AD patients there is an increased rate of hippocampal degeneration with *APOE4* [8, 9], which correlates with cognitive decline and memory deficits [10, 11]. The role of *APOE* in AD is complex and multifactorial, but there is evidence for an interaction with Aβ. In general, *APOE4* is associated with higher Aβ levels and greater markers neuronal dysfunction in the hippocampus compared to *APOE3 in vivo* [12, 13]. In terms of activity, human data are mainly from younger individuals or non-AD patients and indicate higher hippocampal activity with *APOE4* when assessed using fMRI [14–17]. Data on hippocampal glutamatergic activity *in vivo* has focused on the independent effects of *APOE* and human Aβ, and conflict. In fact, both higher and lower hippocampal output/glutamatergic activity have been found in familial AD models (FAD) that overproduce Aβ [18–20] and with *APOE4 in vivo* [11, 21–24]. Importantly, the extent that *APOE* modulates neuronal activity in the context of human Aβ is unclear and limited to one study that utilized acute application of Aβ to hippocampal slices [21]. Therefore, evaluating how *APOE* and Aβ modulate hippocampal neuronal activity is important for understanding *APOE4* associated AD risk.

Angiotensin II was initially linked to AD through hypertension, which increases the risk of developing AD by ~ 35% [25, 26]. Subsequent data implied that that angiotensin II may regulate hippocampal function in the absence of hypertension in AD. Higher Angiotensin II [27] and Angiotensin II Type 1 Receptor (AT1R) levels were found in brains of AD patients compared to controls [28–30]. More direct evidence was found *in vivo*, where inhibiting the AT1R with Angiotensin Receptor Blockers (ARBs) resulted in improved neuronal markers and learning and memory in FAD models [31–39]. In our own studies we found that candesartan (an ARB) treatment of mice that express *APOE4* and overproduce Aβ (E4FAD mice) altered hippocampal neuronal markers and improved short-term memory, although magnitude of the behavioral effects were more modest that we were expecting [36]. An important question raised by these studies, is what is the role of angiotensin II in hippocampal neuron function in the context of *APOE4* and Aβ? Angiotensin II has been shown to impact neuronal activity in the hypothalamus/brainstem, supporting its potential to directly modulate activity [40–45]. Addressing this question is important for advancing our mechanistic understanding of angiotensin II in the brain and its potential role in AD.

Therefore, the goal of this study was to evaluate the role of *APOE* and angiotensin II on hippocampal neuron function in the context of human Aβ. To this end, we used electrophysiological techniques to evaluate basal synaptic transmission, presynaptic activity, and post-synaptic activity in mice that express human *APOE3* (E3FAD) or *APOE4* (E4FAD) and overproduce Aβ.

## Methods

### Mouse models

All experiments were approved by the Institutional Animal Care and Use Committee at the University of Illinois at Chicago. EFAD mice were produced by crossing either *APOE3*- or *APOE4*-targeted replacement mice with mice that express 5 Familial Alzheimer’s Disease (5xFAD) mutations (APP K670N/M671L + I716 V + V717I and PS1 M146L + L286 V) [46]. Both female and male mice (equal numbers) were used and identified by genotyping of tail samples.

### Tissue processing

Mice were anesthetized with 100 mg/kg ketamine and 10mg/kg xylazine (i.p) followed by transcardial perfusion using ice-cold cutting solution (in mM: 93 NMDG, 2.5 KCl, 1.2 NaH_2_PO_4_, 30 NaHCO_3_, 20 HEPES, 10 MgSO_4_, 0.5 CaCl_2_, 25 d-glucose, 5 Sodium Ascorbate, 3 Sodium Pyruvate). The hippocampus was dissected, sectioned (300μm) with a vibratome, and allowed to recover in artificial cerebrospinal fluid (aCSF, in mM: 122 NaCl, 30 NaHCO_3_, 3 KCl, 1.25 NaH_2_PO_4_, 1 MgSO_4_, 10 d-glucose, and 2 CaCl_2_) for 30 minutes at 32^ο^C, bubbled with 95% O_2_ -5% CO_2_.

### Input Output Functions

Input output (I/O) response curves were generated on hippocampal slices prior to induction of high frequency stimulation protocols. Slices were placed in a humidified recording chamber and continuously perfused with aCSF. A glass recording electrode (filled with aCSF) was placed over the apical dendritic layer of CA1 pyramidal neurons and Schaffer collaterals were stimulated using short current pulses delivered with a bipolar electrode roughly 300uM apart. I/O curves were generated using stimulus intensities ranging from 0–200μA in increments of 50μA. Five fEPSPs per stimulus intensity were collected and averaged. fEPSPs for all electrophysiological experiments were recorded and analyzed using AxoGraph software.

### Long-Potentiation Term

Long-term potentiation (LTP) analysis was conducted on hippocampal slices as described previously [47–49] with slight modifications. Basal synaptic transmission was recorded with single stimuli at 50% population spike threshold (ranging from 5–99uA) every 15 s until stable values were obtained for 10 minutes. LTP was induced by a single train of high frequency stimulation (100Hz, 1s at test intensity) and recorded for an additional 30 minutes. For post tetanic potentiation (PTP) analysis, fEPSP amplitudes recorded from 1–3 min after high frequency stimulation were averaged and expressed as a percentage of the average amplitude from 10 minutes of pre-tetanus (baseline) recordings. For LTP analysis, fEPSP amplitudes recorded from 25–30 min after high frequency stimulation were averaged and expressed as a percentage of the average amplitude from 10 minutes of pre-tetanus (baseline) recordings. For time course data, a bin size window of 1 minute was used (i.e., mean value from 4 field responses per data point).

### Paired Pulse Facilitation

Paired Pulse Facilitation (PPF) analysis was conducted on hippocampal slices as described previously, with slight modifications [50]. Two stimuli were applied to the Schaffer collaterals at an interval of 50ms. Paired Pulse Facilitation was determined by taking the ratio of the fEPSP amplitude following the second stimulus to the fEPSP amplitude following the first stimulus (referred to as the paired pulse ratio). For between subject experiments, ten pairs of stimuli were recorded and averaged for analysis. For within subject experiments, 10 minutes of baseline recordings (one pair of stimuli every 15s) were collected prior to bath application of either 10uM Angiotensin II or vehicle followed by an additional 10 minutes of recordings. For time course data analysis, a bin size window of 1 minute was used (i.e., mean value from 4 field responses per data point).

### Statistical Analysis

All data are presented as mean +/− S.E.M and were analyzed by Student’s *t*-test, One-Way ANOVA, or Two-Way ANOVA using Graphpad Prism.

## Results

The goals of this study were 1) to evaluate the role of *APOE* genotype in hippocampal neuron function and then 2) determine the effect of angiotensin II. We used EFAD mice to address these goals, as they express human *APOE3* (E3FAD) or *APOE4* (E4FAD) and overproduce human Aβ, through the expression of 5xFAD autosomal dominant mutations [46]. We used 6-month-old EFAD mice to focus on early/intermediate stages of changes in hippocampal function, since at this age there is greater Aβ plaque accumulation with *APOE4* and the beginnings of behavioral impairments.

### Lower basal synaptic transmission but higher paired pulse facilitation with APOE4 compared to APOE3

**Figure 1:**
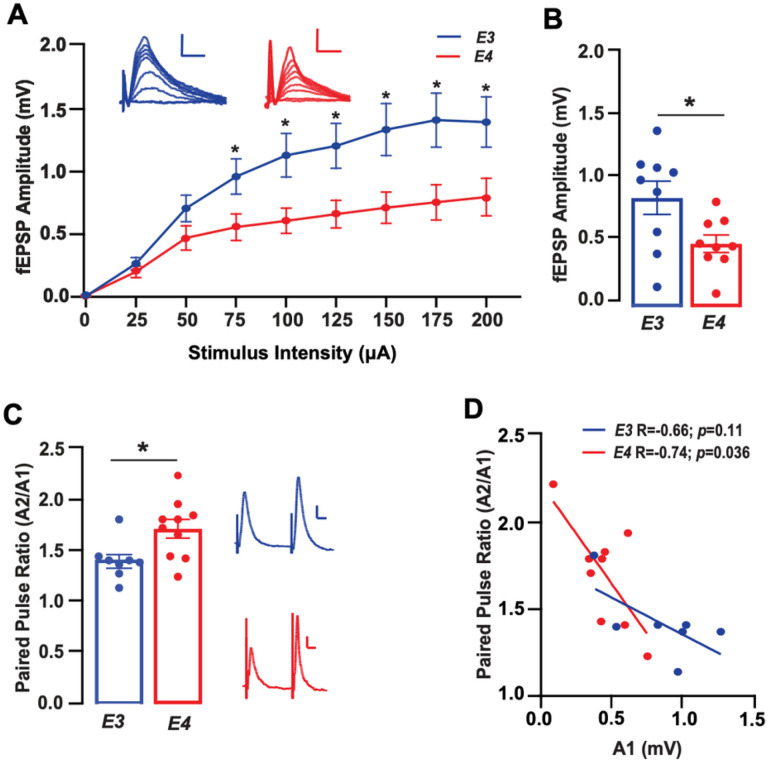
Lower basal synaptic transmission but higher paired pulse facilitation with *APOE4*as compared to *APOE3* **(A)** Input output (I/O) functions for E3FAD and E4FAD mice at stimulus intensities ranging from 0–200μA. Evoked fEPSP amplitudes were lower in E4FAD as compared to E3FAD mice at stimulus intensities > 75mA (*p*<0.05 by *t*-test at each stimulus intensity). **(B)** Two stimuli were applied to the SCCP at 20Hz and amplitudes of resulting fEPSPs were measured to assess paired pulse facilitation (PPF). The Amplitude of the first fEPSP (A1) was lower in E4FAD mice as compared to E3FAD mice (*t*(16)=2.45, *p*=0.023), **(C)** but the paired pulse ratio (Amplitude of the second fEPSP as a ratio of the amplitude of the first fEPSP – A2/A1) was higher in E4FAD as compared to E3FAD mice (*t*(16)=2.54, *p*=0.022). **(D)** There was also a significant correlation between A1 and PPF ratio in E4FAD (*r*=0.74, *p*=0.036) but not E3FAD (*r*=0.66, *p*=0.11) mice. Insets A and C show representative traces from E3FAD and E4FAD mice; calibration bars = 0.2mV, 10ms. All data expressed as mean +/− SEM. *p < 0.05 by *t*-test. *n*=7 for E3FAD mice, and *n*=9 for E4FAD mice.

As the role of *APOE* and Aβ in neuronal function is unknown, we first evaluated basal synaptic activity. This first step is important for determining the alterations in hippocampal function at the synaptic level. We therefore generated Input output (I/O) functions by varying direct synaptic stimulation (input) and measuring the magnitude of the resulting synaptic responses (output) in synapses of the Schaffer Collateral Commissural Pathway (SCCP) in the Stratum Radiatum of the CA1 in E3FAD and E4FAD mice. We found that the magnitude of synaptic responses in E4FAD mice were 30–50% lower than those of E3FAD mice at current inputs ≥ 75μA (Fig. 1A). These types of changes can indicate [51, 52] that there is a lower probability of neurotransmitter release with *APOE4* as compared to *APOE3*. To test this, we next examined paired pulse facilitation (PPF), a form of short-term plasticity critical to information transfer and neural processing [52, 53]. PPF involves a transient increase in the probability of neurotransmitter release during the second of two rapidly evoked responses, an effect which can be quantified as a ratio of the second response relative to the first (A2/A1 – PPF ratio) [51]. However, since this type of facilitation is largely based on the initial probability of release, we began by assessing the magnitude of the first response (A1) on its own at a fixed stimulus test intensity of 50% of population spike threshold. In keeping with our I/O data, we found that A1 was 43% lower in E4FAD mice as compared to E3FAD mice (Fig. 1B). Interestingly, the PPF ratio in E4FAD mice was 22% higher than in E3FAD mice (Fig. 1C), indicating a higher degree of overall facilitation. Higher PPF ratio in E4FAD mice may be due, at least in part, to a lower baseline probability of neurotransmitter release with *APOE4*. In support of this idea, we found that A1 was negatively correlated with the PPF ratio in E4FAD, but not E3FAD, mice (Fig. 1D). Collectively, these data demonstrate that *APOE* genotype modulates basal synaptic transmission by decreasing neurotransmitter release probability.

### Larger magnitude of response to high frequency stimulation with APOE4 as compared to APOE3

**Figure 2:**
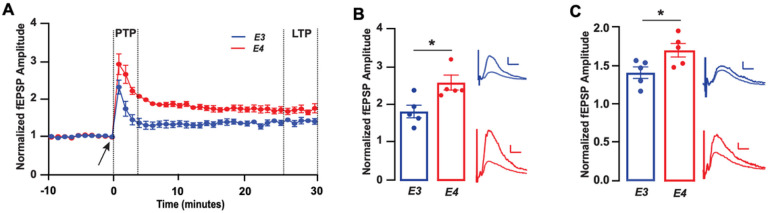
Larger magnitude of response to high frequency stimulation with *APOE4* compared to *APOE3* **(A)** Time course data depicting the effect of high frequency stimulation (HFS) on the amplitude of fEPSPs in E3FAD and E4FAD mice. The arrow at 0 minute indicates induction of HFS protocol. Dashed lines between minutes 1–3 indicate post tetanic potentiation (PTP) period and dashed lines between minutes 25–30 indicate long term potentiation (LTP) period. Amplitude of fEPSPs were higher in E4FAD as compared to E3FAD mice during **(B)** PTP (*t*(8)=2.95, *p*=0.018), and **(C)**LTP (*t*(8)=2.77, *p*=0.024) time periods. Insets B and C show representative traces from E3FAD and E4FAD mice during the PTP and LTP time periods, respectively; calibration bars = 0.1mV, 10ms. All data expressed as mean +/− SEM. *p < 0.05 by *t*-test. *n*=5 for E3FAD mice, and *n*=5 for E4FAD mice.

We next looked at more persistent forms of synaptic plasticity. Long-term potentiation (LTP), thought to be a cellular basis of learning and memory [54, 55], is a form of synaptic strengthening that occurs following a train of high frequency stimulation (HFS). In general, synaptic responses following HFS can be separated into two components: post-tetanic potentiation (PTP) or short-term potentiation [56, 57], which is principally mediated by presynaptic mechanisms, and long-term potentiation (LTP), which is mediated by alterations in glutamate receptors at the postsynaptic site [58, 59]. Therefore, we analyzed PTP and LTP separately to gain a more complete understanding of the true synaptic phenotype of E3FAD and E4FAD mice (Fig. 2A). We began by analyzing PTP and found that the magnitude of response was 30% higher in E4FAD as compared to E3FAD mice (Fig. 2B). Similarly, when we analyzed LTP, we found that potentiation levels were 20% higher in E4FAD mice as compared to E3FAD mice (Fig. 2C). Together these data suggest a change in basal release probability and a higher magnitude of presynaptic *and* postsynaptic response to high frequency stimulation with *APOE4* as compared to *APOE3*.

### No effect of angiotensin II on basal synaptic transmission or synaptic facilitation with APOE3 or APOE4

**Figure 3:**
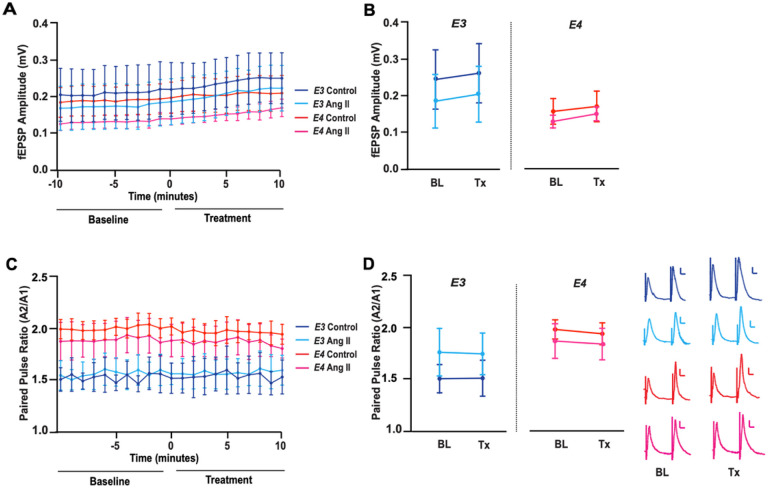
No effect of angiotensin II on basal synaptic transmission or synaptic facilitation with *APOE3* or *APOE4*. **(A)** Paired stimuli were applied to the SCCP at 20 Hz every 15s for 20 minutes to assess basal synaptic transmission and paired pulse facilitation (PPF). Time course data of the amplitude of the first response (mV) is depicted. Time 0 indicates bath application of either 10uM of angiotensin II or vehicle treatment. **(B)** Exogenous angiotensin II did not alter the amplitude of the first evoked fEPSP (A1) in E3FAD (*t*(8)=0.18, *p*=0.86) or E4FAD (*t*(8)=0.81, *p*=0.43) mice. Vehicle treatment also did not alter A1 in E3FAD (*t*(6)=0.15, *p*=0.88) or E4FAD (*t*(8)=0.27, *p*=0.79) mice. **(C)**Time course data of the paired pulse facilitation ratio (A2/A1) is depicted. **(D)**Exogenous angiotensin II did not alter the PPF ratio in E3FAD (*t*(8)=0.053, *p*=0.96) or E4FAD (*t*(8)=0.13, *p*=0.89) mice. Vehicle treatment also did not alter the PPF ratio in E3FAD (*t*(6)=0.022, *p*=0.98) or E4FAD (*t*(8)=0.30, *p*=0.77) mice. All data expressed as mean +/− SEM. *p* > 0.05 by *t*-test. *n* =4 for E3FAD vehicle, *n* =5 for E3FAD Ang II, *n*=5 for E4FAD vehicle, and *n*=5 for E4FAD Ang II.

We next evaluated the impact of angiotensin II on the *APOE* modulated synaptic response. We started with examining the effect of exogenous angiotensin II on basal synaptic transmission in the SCCP measured similarly to above in E3FAD and E4FAD mice (Fig. 3A) and found that the magnitude of the synaptic response did not change in either genotype (Fig. 3B). We also analyzed the effect of angiotensin II on paired pulse facilitation (Fig. 3C) and found that the paired pulse ratio also did not change in either E3FAD or E4FAD mice (Fig. 3D). These data imply that exogenous angiotensin II does not modulate basal synaptic transmission or paired pulse facilitation in the SCCP of EFAD mice.

### Angiotensin II suppresses the magnitude of response to high frequency stimulation with APOE3 and APOE4

**Figure 4:**
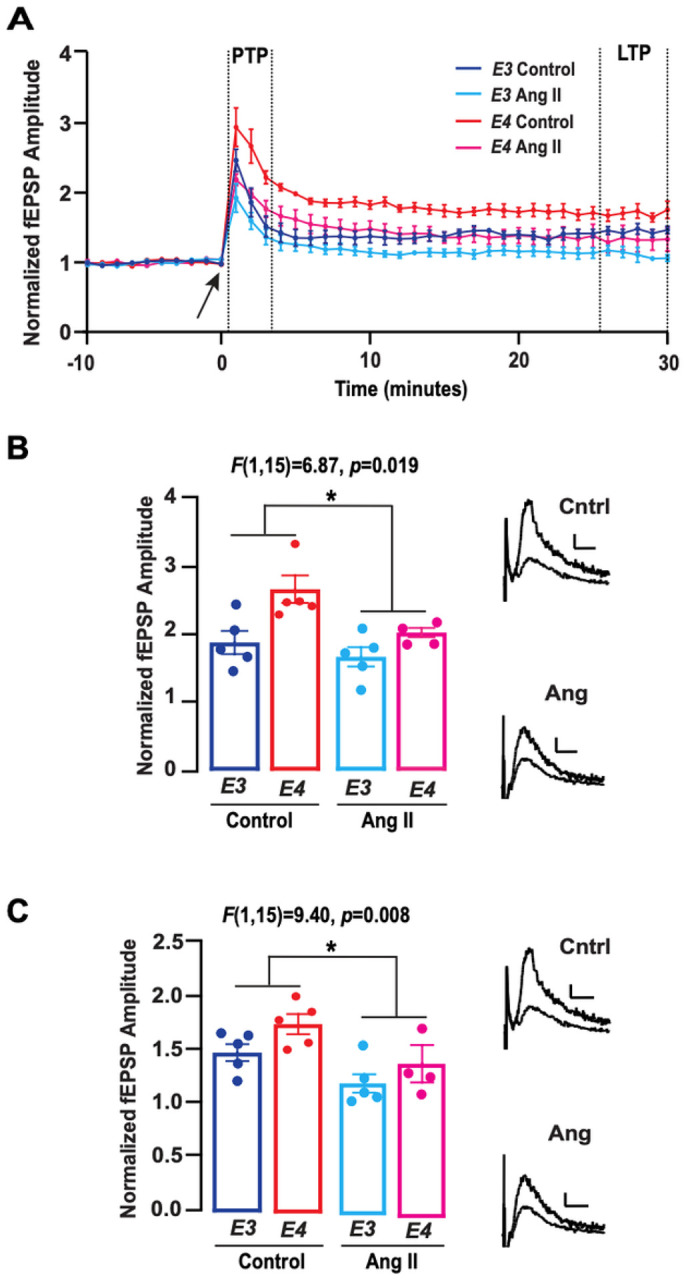
Angiotensin II suppresses the magnitude of response to high frequency stimulation with *APOE3*and *APOE4*. **(A)** Time course data depicting the effect of high frequency stimulation (HFS) on the amplitude of fEPSPs in E3FAD and E4FAD mice. The arrow at 0 minute indicates induction of HFS protocol. Dashed lines between minutes 1–3 indicate post tetanic potentiation (PTP) period and dashed lines between minutes 25–30 indicate long term potentiation (LTP) period. **(B)** During the PTP period, amplitude of the fEPSPs were lower with the addition of 10uM angiotensin II in E3FAD and E4FAD mice (*F*(1,14) = 5.38, *p*= 0.036). **(C)** During the LTP period, amplitude of the fEPSPs were also lower with the addition of 10uM angiotensin II in E3FAD and E4FAD mice (*F*(1,14) = 11.06, *p* = 0.005). Insets B and C show representative control and angiotensin II traces for both the PTP and LTP periods. Calibration bars = 0.1mV, 10ms. All data expressed as mean +/− SEM. *p < 0.05 by two-way ANOVA.

Finally, we assessed whether angiotensin II would impact longer term, NMDA-receptor dependent forms of plasticity. To this end, we evaluated the effects of angiotensin II on the magnitude of synaptic responses to high frequency stimulation in E3FAD and E4FAD mice (Fig. 4A). We first analyzed PTP and found that for both genotypes, the magnitude of response was ~ 20% lower with angiotensin II as compared to control (Fig. 4B). Likewise, with LTP, we found that the magnitude of response was ~ 20% lower with angiotensin II as compared to control for both genotypes (Fig. 4C). Taken together this data suggests that while angiotensin II does not modulate hippocampal short-term plasticity, it does impact longer-term forms of plasticity. Effects on PTP suggest a presynaptic effect while those on LTP imply a potential post-synaptic mechanism of action.

## Discussion

### APOE4 and Neuron Function

Identifying how known AD-risk factors impact neuronal activity is important for our understanding of the disease, and here we found *APOE4* and high Aβ levels are associated with lower basal synaptic transmission and greater responses to high frequency stimulation (HFS) in the hippocampus. This phenotype is in partial agreement with pervious human and *in vivo* studies. In non-AD context, compared to *APOE3*, higher hippocampal activity has been found with *APOE4* in several studies using fMRI [14–17]. However, age and AD status may impact the extent that *APOE4* differs from *APOE3*. For example, it has been suggested that higher hippocampal activity represents a feature of cognitive impairment for all *APOE* genotypes [60], or alternatively that hippocampal activity is lower with age with *APOE3* but not *APOE4* carriers [61]. Somewhat related is the higher association of *APOE4* with seizures and epilepsy that implies network hyperexcitability is a general feature with *APOE4* in humans [62–65]. Taken together these human studies broadly imply higher hippocampal activity with *APOE4* as compared to *APOE3*, with the caveat that age, disease severity and region are important considerations. Data from mouse models highlights a complex interaction with *APOE* genotype and hippocampal activity. In the dentate gyrus/medial-perforant pathway [11, 21] there is lower LTP induction and maintenance with *APOE4* compared to *APOE3* in young mice. However, there is no difference between *APOE* genotypes in old mice in the same circuit, data that implies the early changes are negated due to age-related impairments in hippocampal plasticity [66, 67]. In layers II/III of the entorhinal cortex, higher spontaneous glutamatergic neuronal transmission has been found with *APOE4* compared to *APOE3* that with age lead to lower potentiation following HFS [68]. In the CA1/SCCP and similar to our findings, LTP responses are generally higher with *APOE4* [22–24] in young mice, although a few have also reported lower responses [69, 70]. Thus, in the absence of high Aβ, the impact of *APOE* appears to be dependent on brain circuit and age. Future studies could focus on clarifying the overall impact of *APOE4*, brain region and age on hippocampal activity. In general, however, there is some consensus that for the CA1 *APOE4* is associated with hippocampal hyperactivity, however whether this is impacted by high levels of Aβ is unknown.

High Aβ levels are a major pathological hallmark of AD and therefore may interact with *APOE* to modulate hippocampal activity. In models of high Aβ caused by overexpression of familial AD (FAD) mutations, data are conflicted as to the effects of chronic exposure to Aβ on hippocampal electrophysiology. For example, there are reports of age-dependent reductions in LTP in the CA1/SCCP [71–74] and in the dentate gyrus/perforant pathway [75–78] of various FAD mouse models including 5xFAD [74]. Conversely, there is also evidence indicating transient enhancements in hippocampal activity in the CA/SCCP [79–82] and in the dentate gyrus [83]. In terms of LTP responses with Aβ and *APOE*, there is only one report on the role of exogenously added oligomeric Aβ in young mice that express the human *APOE* gene. Those data demonstrate an isoform-specific inhibitory effect on hippocampal neuronal activity in the medial perforant pathway following the order *APOE4* > *APOE3* > *APOE2* [21]. However, in our model system we show that chronic high levels of Aβ with *APOE4* is associated with enhanced levels of LTP in the CA1/SCCP. The differences between data may be related to the circuit (perforant vs CA1/SCCP), age, and/or model (chronic vs acute). Future studies could address how these factors affect the interaction of *APOE4* and Aβ on neuron activity.

Our data raise the important question of what higher hippocampal neuron activity in the CA1/SCCP may mean in the context of AD. One possibility is that higher activity is a general property associated with *APOE4* across the lifespan and has no impact on neural circuit disruption of cognitive dysfunction in AD. The other extreme is that hyperactivity is a detrimental or maladaptive response due to higher Aβ levels and/or the response of *APOE4* to Aβ. There are also several alternatives to these extremes. For example, as Aβ accumulation can have inhibitory effects on hippocampal activity (discussed above), heightened activity in the SCCP with *APOE4* may be an important compensatory mechanism early on in disease progression to preserve neural output. Conversely, high levels of hippocampal activity with *APOE4* may represent an example of antagonistic pleiotropy, a function that is beneficial early in life, but detrimental later. Evidence for the *APOE4* antagonistic pleiotropy hypothesis comes from studies conducted in young *APOE4*-carriers that outperform non-carriers on memory and neurocognitive tasks early in life, potentially due to greater involvement of executive processes [84, 85]. The idea is that due to continuous higher activity, the circuit is predisposed to dysfunction in AD; or, to compensate for declines in older age, this same recruitment mechanism leads to detrimental hippocampal hyperactivity, ultimately contributing to accelerated cognitive decline. Consistent with this, is the idea that lowering hippocampal excitability levels with *APOE4* may be beneficial in AD. Indeed, preventing hyperexcitability has been documented to improve memory performances in AD transgenic mice [86, 87] most likely by enhancing responsiveness to GABAergic interneuron inputs [68, 88]. Future studies will ultimately reveal to what extent *APOE4*-driven hyperactivity may be a contributing factor to increased AD risk.

Our data also raise the question of what potential mechanisms may underlie the altered hippocampal activity with *APOE4* and Aβ. In general, the question of how *APOE* impacts neuronal function is considered pleiotropic including modulating neuronal function directly and indirectly. As broad examples, *APOE4* is associated with greater neurovascular dysfunction, metabolic dysfunction, neuroinflammation and peripheral inflammation, processes that independently can all disrupt neuronal activity [7, 89]. There are also specific neuronal mechanisms that are disrupted with *APOE4* including inhibitory network function within the hippocampus (reviewed in [88]). For example, in *APOE*-targeted replacement mice, compared to *APOE3*, with *APOE4* there are lower levels of GABAergic somatostatin positive interneurons in the hippocampus, an effect that appears driven by apoE production in neurons [88]. Thus, the loss of GABAergic interneurons could contribute to network hyperexcitability and higher levels of pyramidal cell firing [68]. Another possibility is the idea that apoE4 derived specifically from astrocytes enhances neuronal excitability [90], potentially due to lysosome dysregulation, altered membrane lipidomes, and/or Ca^2+−^induced hyperactivity [91]. Collectively all these factors could contribute to the phenotype we found in hippocampal neurons of lower basal synaptic transmission combined with enhanced PPF and LTP.

At the cellular level in glutamatergic neurons, our data suggests that the impact of *APOE4*, either due to the mechanisms described above or others, causes changes in both the presynapse and post synapse. In the presynapse, we found lower magnitudes of evoked fEPSPs combined with enhanced PPF ratios with *APOE4* compared to *APOE3*. This phenotype could be caused by dysregulation in presynaptic calcium homeostasis with *APOE4* [92, 93]. In AD, neurons tend to have higher levels of resting calcium which has been attributed to enhanced calcium entry and/or enhanced calcium leakage from intracellular stores [94]. If there are higher neuronal calcium levels with *APOE4* due to calcium leakage and/or buffering, it would mean that baseline neuronal activity would be lower because it would interfere with membrane depolarization and thus the probability of firing action potentials. In addition, repeated stimulation (i.e., tetanus), would trigger the release of abnormally high levels of intracellular calcium from organelles such as the mitochondria and endoplasmic reticulum with *APOE4*. This would, in turn, result in a higher number of neurotransmitter-containing vesicles to fuse with the plasma membrane, thereby increasing presynaptic glutamate release resulting in higher levels of responses to high frequency stimulation, in agreement with our data. Relatedly, it has been proposed that *APOE* modulates the glutamate-glutamine cycle, in that with *APOE4* there is lower glutamate production and ultimately less efficient vesicular loading [24, 95]. Consistent with this, our PPF data support a lower probability of neurotransmitter release as part of the *APOE4* phenotype which could be explained by lower glutamate production, less efficient loading of glutamate into synaptic vesicles, and/or dysfunctions in the presynaptic vesicular fusion/release mechanisms (potentially due to calcium buffering deficits). Due to any combination of these factors, higher levels of presynaptic input may be required with *APOE4* to elicit the same postsynaptic responses as *APOE3* under basal conditions. We have also provided direct evidence that *APOE4* modulates post synaptic neuronal signaling mechanisms. In keeping with other reports [23], we observed a substantially larger magnitude of response to high frequency stimulation with *APOE4* as compared to *APOE3*. Higher post synaptic activity can be caused by changes in AMPA and NMDA composition, levels, and signaling. In terms of *APOE*, most data on post synaptic mechanisms are related to receptor signaling. ApoE4 is thought to enhance ERK1/2 activation through interactions with the LRP1 receptor which promotes induction of LTP to a greater extent than apoE3 [23, 96]. It has also been reported that *APOE4* suppresses LTP induced by reelin due to modulating glutamate receptor phosphorylation and/or sequestration [24, 95]. The lower response to reelin *in vivo* could cause a compensatory upregulation response with *APOE4* and Aβ. Therefore, with *APOE4* there could be changes at the post synapse in signaling, receptor levels, or calcium responses [79] that result in greater LTP responses following tetanic stimulation. A final explanation for our data is lower overall GABAergic inputs to CA1 neurons, resulting in a heightened response to repeated glutamatergic inputs manifesting in aberrantly increased hippocampal activation with *APOE4* [88]. Future mechanistic studies could inform how alterations in presynaptic and postsynaptic signaling with *APOE* genotype modulate hippocampal circuitry in AD.

### Angiotensin II and Neuron Function

We found that angiotensin II suppresses neuronal activity, which raises the important question of the significance of this finding in the context of AD and *APOE4*. In general, higher angiotensin II levels and /or receptor signaling are considered detrimental in AD [29, 38, 97, 98]. This proposal is based on data that in the medial frontal cortex of AD patients there are up 40% higher angiotensin II levels [27] as well as higher ACE and AT1R levels in the hippocampus and prefrontal cortex as compared to age matched controls [28–30]. Further, AT1R levels are 2.5x higher in the hippocampus [99] and 3x higher in the cortex [100] of APPJ20 mice as compared to wild type controls. In support that enhanced levels of angiotensin II is detrimental for brain function are findings that blocking the AT1R is beneficial in FAD mouse models [31–39]. Specific to *APOE4*, we found a slight improvement in behavior in EFAD mice after ARB treatment [36]. However, caution may be warranted in assigning a beneficial vs. detrimental impact of angiotensin II to brain function, including with *APOE4*. Angiotensin II binds receptors on multiple cell types including glia and endothelial cells to exert pleotropic mechanisms of action. In fact, in many *in vivo* studies, including ours in E4FAD mice, the strongest effect of ARB treatment appears to be preventing enhanced glial activation and modulating neuroinflammatory markers due to high Aβ levels. However, despite a strong effect on glia in our previous study, the corresponding change in behavior was relatively modest in E4FAD mice. This raises the possibility that if higher hippocampal output is detrimental for *APOE4* carriers, then angiotensin II dependent suppression may be beneficial, and therefore blocking the AT1R globally is not optimal. Alternatively, if higher LTP is a beneficial compensatory mechanism, then preventing the angiotensin II dependent suppression of LTP is optimal. Therefore, there may also be a balance, whereby neither too low nor too high levels of LTP are optimal for both *APOE3* and *APOE4*, and therefore maintaining a certain moderate level of LTP is more important. Interestingly, while use of angiotensin system blockers was associated with slower global Aβ accumulation over time and a lower incidence of AD in *APOE4*-non carriers, this effect was not seen in *APOE4* carriers [101, 102]. Ultimately, understanding how the fundamental cell-type specific functions of angiotensin II/AT1R collectively contribute *in vivo* to behavior is important for a deeper mechanistic and therapeutic understanding of the angiotensin system in AD. Recognizing the complexity of AD, the relatively contribution of AT1R on each cell type may depend on the stage of AD and the relative contribution of inflammation, vascular dysfunction and neuron hyperactivity to cognitive impairment in each patient.

Mechanically, our data supports that while exogenous angiotensin II does not impact basal synaptic transmission or neural facilitation, it does have a profound inhibitory effect on hippocampal LTP in mice that express human *APOE*. In general, data are mixed on the role of angiotensin II on neuronal excitability with reports of both excitatory and inhibitory effects at the single cell level depending on the brain region and neuronal subpopulation [42, 103, 104]. However, our LTP result is in agreement with other studies demonstrating the inhibitory effects of angiotensin II on synaptic plasticity including in the medial perforant pathway [105] and the lateral nucleus of the amygdala [106]. The majority of the functions associated with angiotensin II signaling in neurons are mediated through AT1R signaling pathways. The AT1R is a G-protein coupled receptor of the G_αq_ subtype. G_αq_ receptors activate Protein Kinase C (PKC), which regulates calcium-dependent inactivation of NMDA receptors [107, 108]. Lower levels of NMDA receptor activation at the post synapse would lead to a lower responsiveness to glutamate and therefore suppression of LTP, consistent with our data. Taken together, this suggests that high levels of AT1R activation with angiotensin II interferes with NMDA receptor-dependent synaptic plasticity in the SCCP.

## Conclusions

Collectively, our data suggests that *APOE4* and Aβ are associated with a hippocampal phenotype comprised of lower basal activity and higher stimulus evoked responses, the latter of which is suppressed by angiotensin II. These novel data suggest a potential mechanistic link between hippocampal activity, *APOE4* genotype and angiotensin II in AD.

## Data Availability

The datasets used and/or analyzed during the current study are provided as a supplementary file and are available from the corresponding author on reasonable request.

## Abbreviations

AD: Alzheimer’s disease
ARBs: Angiotensin Receptor Blockers
AT1R: Angiotensin Type 1 Receptor
aCSF: Artificial cerebrospinal fluid
EFAD mice: mice that express human *APOE3*- or *APOE4*-and 5 FAD mutations (APP K670N/M671L + I716 V + V717I and PS1 M146L + L286 V)
FAD: Familial AD models
fEPSPs: Field excitatory postsynaptic potential
HFS: High frequency stimulation
LTP: Long-term potentiation
PPF: Paired pulse facilitation
PTP: Post-tetanic potentiation
SCCP: Schaffer Collateral Commissural Pathway
